# Strategies for Alleviating Electrode Expansion of Graphite Electrodes in Sodium‐Ion Batteries Followed by In Situ Electrochemical Dilatometry

**DOI:** 10.1002/ente.202000880

**Published:** 2020-11-19

**Authors:** Ines Escher, Yuliia Kravets, Guillermo A. Ferrero, Mustafa Goktas, Philipp Adelhelm

**Affiliations:** ^1^ Institut für Chemie Humboldt Universität zu Berlin Brook‐Taylor‐Str. 2 12489 Berlin Germany; ^2^ Joint Research Group Operando Battery Analysis Helmholtz‐Zentrum Berlin Hahn‐Meitner‐Platz 1 14109 Berlin Germany

**Keywords:** electrochemistry, in situ electrochemical dilatometry, pillared graphite, sodium‐ion batteries, solvated co‐intercalations

## Abstract

The electrochemical intercalation/deintercalation of solvated sodium ions into graphite is a highly reversible process, but leads to large, undesired electrode expansion/shrinkage (“breathing”). Herein, two strategies to mitigate the electrode expansion are studied. Starting with the standard configuration (−) sodium | diglyme (2G) electrolyte | graphite (poly(vinylidene difluoride) (PVDF) binder) (+), the PVDF binder is first replaced with a binder made of the sodium salt of carboxymethyl cellulose (CMC). Second, ethylenediamine (EN) is added to the electrolyte solution as a co‐solvent. The electrode breathing is followed in situ (operando) through electrochemical dilatometry (ECD). It is found that replacing PVDF with CMC is only effective in reducing the electrode expansion during initial sodiation. During cycling, the electrode breathing for both binders is comparable. Much more effective is the addition of EN. The addition of 10 v/v EN to the diglyme electrolyte strongly reduces the electrode expansion during the initial sodiation (+100% with EN versus +175% without EN) as well as the breathing during cycling. A more detailed analysis of the ECD signals reveals that solvent co‐intercalation temporarily leads to pillaring of the graphite lattice and that the addition of EN to 2G leads to a change in the sodium storage mechanism.

## Graphical abstract

The electrode expansion and shrinkage (“breathing”) of a graphite electrode in a sodium‐ion battery is investigated. Changing the binder material from poly(vinylidene difluoride) to the sodium salt of carboxymethyl cellulose results in a smaller electrode breathing in the first cycle but does not influence the following cycles. The addition of ethylenediamine as co‐solvent results in reduced breathing in all cycles.

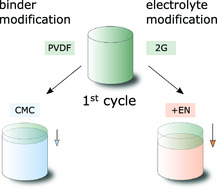

## 1 Introduction

Sodium‐ion batteries (SIBs) are currently considered as cost‐effective and more sustainable alternatives to lithium‐ion batteries (LIBs).^[^ ^1^ ^]^ Graphite, due to its frequent use in LIBs, is naturally also of interest for SIBs. However, intercalation of sodium ions into graphite is thermodynamically unfavorable;^[^ ^2^ ^]^ hence, the storage capacity is very small. A way around this problem is to co‐intercalate solvent molecules to form so‐called ternary graphite intercalation compounds (t‐GICs). t‐GICs have been known for decades, but it was only in 2014 that the reversible intercalation of “solvated sodium ions” into graphite over many cycles was reported using 2G as the electrolyte solvent.^[^ ^3^ ^]^ The following reaction takes place

(1) $$C_{n}+e^{-}+A^{+}+y\;\text{solv}⇌A^{+}{\left(\text{solv}\right)}_{y}C_{n}^{-}$$

where the metal ion (A^+^) is intercalated together with solvent molecules (solv) into the graphite lattice (C). The reaction does also take place for A^+^ = Li^+^,^[^ ^3^, ^4^ ^]^ but the high‐current behavior is poor due to the strong lithium–graphite interaction. As a result of this, the lithium ions become desolvated and the remaining free solvent molecules hinder the ion movement within the graphite lattice.^[^ ^5^ ^]^ During the aforementioned reaction, graphite is reduced and a specific number of solvent molecules (solv) per alkali metal ion (A^+^) is intercalated.^[^ ^6^ ^]^ The ratio is still not completely clarified, but values of 1–2 and 15–22 for *y* and *n* are assumed.^[^ ^2^, ^3^ ^−^ ^4^, ^7^ ^]^ The reaction has some characteristic properties: 1) the capacity so far is limited to around 110 mAh g^−1^,^[^ ^8^ ^]^ which is low compared with conventional battery electrodes (typically >150 mAh g^−1^), but high compared with supercapacitor electrodes;^[^ ^9^ ^]^ 2) the initial Coulomb efficiency (ICE) is very high (≈ 90% or even higher^[^ ^3^, ^7^ ^]^); 3) the rate capability and cycle life are excellent (up to 6000 cycles with 100 mAh g^−1^^[^ ^10^ ^]^); 4) the concept requires an “SEI‐free” interface,^[8a]^ and 5) the volume expansion/shrinkage during cycling is very large (70–100%).^[8a]^

This article addresses the last aspect, i.e., the volume expansion during cycling. On a material level, the graphite interlayer spacing increases from 3.35 to 11.6–12.0 Å during intercalation, i.e., by about 250–260%.^[7b−d]^ On the other hand, expansion on the electrode level is notably smaller. Our group has previously observed the electrode expansion/shrinkage (“breathing”) to be about 70–100% during cycling using in situ electrochemical dilatometry (ECD).^[8a]^ The difference is caused by the electrode porosity which partially buffers the volume changes of the active materials; see also Sn/carbon composites as an example.^[^ ^11^ ^]^ Note that the ECD device continuously measures the thickness change of an electrode during cycling. Although the technique is often mentioned as an in situ method, it actually operates in operando mode. Karimi et al. used different types of glymes and showed a relative thickness change (expansion or shrinkage) of around 45–85% in the second to fifth cycle. These values converge with increasing cycling number.^[^ ^12^ ^]^

However, despite the excellent cycle life of this type of electrodes and the first results from full cells with various cathodes,[^7^, ^8^, ^10^, ^13^] it is clear that such large volume changes are undesirable and should be minimized.

Here, we report on two strategies to reduce the electrode breathing of graphite electrodes in sodium‐ion cells and study the effectiveness of the approach by ECD over several cycles. First, we study the effect of the type of binder. Varying the type of binder is motivated by the different mechanical properties of poly(vinylidene difluoride) (PVDF) and the sodium salt of carboxymethyl cellulose (CMC) which are the two most commonly used types of binder for LIBs and SIBs.

Second, the influence of the addition of ethylenediamine (EN) as a co‐solvent is investigated. Such use of EN is motivated by recent results from Zhang et al.^[^ ^14^ ^]^ who found a reduction in volume expansion by more than half when adding EN to 2G as a co‐solvent. The authors propose that after desodiation, some solvent molecules remain in the graphite lattice leading to “pillared graphite”. The structural analysis was done by ex situ X‐ray diffraction (XRD), i.e., the crystalline properties after charge and discharge were determined. As discussed earlier, the volume change on the material level is very different from the volume change on the electrode level. The ECD study presented here, therefore, complements the results from Zhang et al. and shows the effectiveness of minimizing electrode breathing by the use of co‐solvents with an in situ (operando) method. In addition, not only the first cycle is investigated but also information about EN upon continuous cycling is gained, revealing that the pillaring effect is only temporary.

## 2 Results and Discussion

Preliminary note: Based on our previous publications,[^8^, ^15^] electrodes prepared with a PVDF binder and cells with 2G as the solvent served as reference. Improvements through changing the type of binder or adding EN as a co‐solvent are given relative to this reference.

### 2.1 Influence of the Binder on Electrode Expansion/Shrinkage

PVDF and CMC are the most common binders used in battery research. Although PVDF is still commonly used to produce carbonaceous as well as noncarbonaceous anodes for SIBs,^[^ ^16^ ^]^ the need for NMP (*N*‐methylpyrrolidone) as the solvent during slurry preparation is undesired due to health and environmental concerns. On the other hand, aqueous processing is possible in case of CMC; this being the reason why water‐soluble binders are preferred and used for commercial production of LIB graphite electrodes.^[^ ^17^ ^]^ The general characteristics of both types of binders and the scanning electron microscopy (SEM) cross‐section images of the prepared electrodes are shown in Table S1, Figure S1 and S2, Supporting Information. The main difference is that PVDF can be found mainly on top of the electrode, whereas CMC is more homogeneously distributed in the volume.

**Figure 1:**
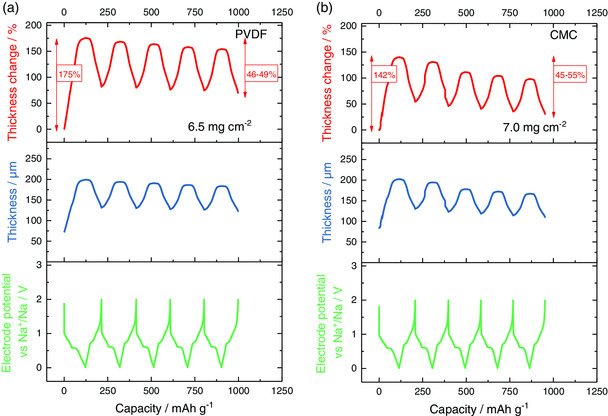
In situ ECD experiments of graphite electrodes with two different binders a) PVDF, b) CMC in a three‐electrode set‐up with sodium as counter and reference electrode, conducted with a C‐rate of 0.1 C (11 mA g^−1^).

The impact of the type of binder on the electrode breathing in sodium‐ion half cells is shown in **Figure** **1**. Note that the type of binder also influences the electrode preparation, which is why there is a slight deviation in electrode loading (active material) and initial thickness (with current collector) (*m*_with CMC_ = 7.0 mg cm^−2^ vs *m*_with PVDF_ = 6.5 mg cm^−2^ and *h*_0,with CMC_ = 97 μm versus *h*_0,with PVDF_ = 85 μm). As can be observed from the voltage profile and the ECD signal, intercalation of sodium ions (cell discharging) leads to an electrode expansion while deintercalation (cell charging) leads to an electrode shrinkage. Figure 1 shows results for the first five cycles.

The obtained capacities range from 95–106 mAh g^−1^, which is close to the theoretical value of around 110 mAh g^−1^ for [Na(2G)_*x*_]C_20_. This means that the electrode capacity does not depend on the type of binder (which is expected and shows that a comparison is feasible). On the other hand, the ECD results show that the type of binder has a notable influence on the electrode expansion during the first cycle. Although the first sodiation leads to an expansion of 175% in the case of PVDF, 142% is found for CMC. The difference in 33 percentage points corresponds to an improvement for the CMC electrode by 19% (33/175) when referring to the PVDF electrode as a reference. During subsequent desodiation, the electrode does not return to its original thickness, which is due to structural rearrangement and exfoliation of the particles leading to larger electrode porosity as previously discussed.^[8a]^ During subsequent cycling, breathing of the electrodes is similar for CMC (45–55%) and PVDF (46–49%).

Overall, the results clearly show that the type of binder has a notable influence on the initial electrode expansion with CMC leading to a smaller increase compared with PVDF. This may be caused by several factors: 1) CMC is more homogeneously distributed over the electrode (see Figure S1 and S2, Supporting Information) which might lead to a better preservation of the electrode structure compared with PVDF; 2) CMC has a higher Young's modulus compared with PVDF and, therefore, a higher stiffness,^[^ ^18^ ^]^ which could counteract more efficiently the expansion of the electrode, and 3) the interactions of CMC with the graphite surface. Regarding the latter, whereas the backbone of CMC interacts with graphite and stabilizes the structure,^[^ ^19^ ^]^ the interactions between hydrogen and fluoride in PVDF are stable and, therefore, the interaction with the graphite surface is weak.^[^ ^20^ ^]^

**Figure 2:**
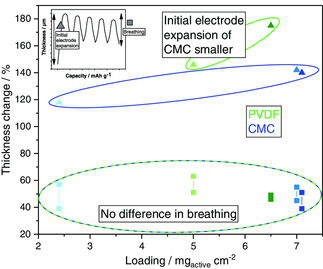
Thickness change of graphite electrodes during intercalation/deintercalation of solvated sodium ions versus loading of the active material. Data obtained with a three‐electrode cell set‐up with sodium as counter and reference electrode. Initial electrode expansion shown with triangles, electrode breathing shown with squares.

In a second series of experiments, the influence of the electrode loading (and with that the initial electrode thickness) was studied; see Figure S3 and S4, Supporting Information. The results are summarized in **Figure** **2** and are in line with the findings shown in Figure 1, i.e., the type of binder influences the first cycle (shown with triangles), where electrodes made with CMC show an overall lower electrode expansion compared with the ones made with PVDF. In the following cycles, breathing of the electrodes is in the same range for both binders. An overview of all experiments conducted with different loadings is shown in Table S2, Supporting Information. As can be observed, the thickness changes in the initial cycle of the graphite electrodes are always lower in the case of the CMC binder. In addition, even a difference in the mass loading as large as 2.1 mg cm^−2^ (*m*_with CMC_ = 7.1 mg cm^−1^ vs m_with PVDF_ = 5.0 mg cm^−1^) (which translates into 39 μm higher initial thickness) leads to a lower thickness change in the first cycle for the CMC‐based electrode compared with the one made with PVDF. Although this study is the first report on binder comparison for solvent co‐intercalation reactions, the findings are in line with previous studies on hard carbon electrodes^[^ ^21^ ^]^ and SnO_2_ electrodes^[^ ^22^ ^]^ for which the use of CMC also leads to better properties compared with PVDF. For comparison, we tested the graphite electrodes also in Li‐ion half cells and found the same trend, i.e., the expansion is reduced in the first cycle in case CMC is used as binder; see Figure S5, Supporting Information.

### 2.2 Influence of EN as a Co‐Solvent on Electrode Expansion/Shrinkage

**Figure 3:**
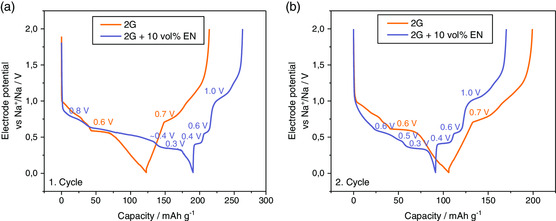
Voltage profiles a) first cycle, b) second cycle of a sodium‐ion cell with graphite (PVDF as binder) as the working electrode in a three‐electrode cell set‐up with sodium as counter and reference electrode. Conducted with 11 mA g^−1^ (corresponds to a C‐rate of 0.1 C for [Na(2G)_*x*_]C_20_). Comparison of pure 2G as the electrolyte solvent (orange) and the addition of 10 v/v EN (purple). The voltage plateaus are labeled.

Very recently, Zhang et al.^[^ ^14^ ^]^ showed through ex situ XRD that the expansion of the graphite lattice during t‐GIC formation can be reduced by 57% when adding EN to a 2G electrolyte. Although only the initial expansion was studied, the approach seems promising. Encouraged by these findings, we studied whether the smaller lattice expansion also translates into reduced electrode breathing during cycling. To that end, graphite electrodes (with PVDF) were cycled in sodium‐ion half cells with electrolyte solutions based on pure 2G and 2G + 10 v/v EN. **Figure** **3** shows the voltage profiles of the graphite cells with and without EN as a co‐solvent. As expected from previous studies, results for the first two cycles are comparable when 2G is used as the electrolyte solvent, i.e., the reaction is reversible. The Coulomb efficiencies of the first (ICE) and second cycle reach 74% and 88%, respectively. This is in fact lower compared to when coin cells are used, but we generally observe somewhat lower ICE values in the ECD device. In contrast, cells made with the addition of EN show a very different behavior. During initial discharging, the plateaus are slightly shifted, especially the middle region, which becomes elongated. In addition, it does not occur as a single plateau, but as a sloping region with a small plateau in the end. The discharge capacity reaches 191 mAh g^−1^ but is only partially regained during charging. The low ICE of 38% indicates a much more severe electrolyte decomposition compared to pure 2G. In the second cycle, the reaction becomes more reversible, reaching a Coulomb efficiency of 87% and a discharge capacity of 91 mAh g^−1^, being slightly lower compared to 2G. In the second to fifth cycle, the Coulomb efficiencies reach values of 88–93% for the pure 2G system and 86–95% when 10 v/v EN is added, showing that both systems have a similar, good reversibility. The low ICE value and the complex storage process are drawbacks of using EN and further investigations on their origin are required.

Importantly, the additional plateaus in the voltage profile remain, meaning that the redox reaction (see Equation (1)) changes. In other words, EN participates in the electrode reaction and changes the formation of the t‐GIC. The change in the redox reaction due to EN is also corroborated through the different XRD patterns of the electrodes after full sodiation, as can be seen in Figure S6, Supporting Information. Thus, when the graphite electrode was fully sodiated in pure 2G, the reflections for the t‐Na‐GIC at 15.3°, 23.8°, and 29.9° correspond to the expected stage 1 t‐GIC.[^7^, ^8^] In contrast, the final product of the sodium electrode with 2G + EN at 0.01 V exhibits a different behavior. The XRD pattern shows the existence of well‐defined signals at 12.6° as well as at 25.5° that can be attributed to a monolayer t‐GIC as suggested by Zhang et al.^[^ ^14^ ^]^ Although a difference in XRD patterns is evident between 2G and 2G + EN, a more clear interpretation of the diffraction date will require in situ studies.

**Figure 4:**
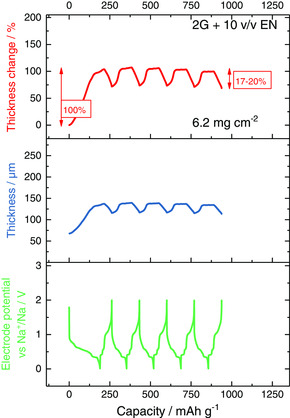
In situ ECD experiments of a sodium‐ion cell with graphite (PVDF as binder) as working electrode in a three‐electrode set‐up with sodium as counter and reference electrode. 1 m NaOTf in 2G + 10 v/v EN is used as electrolyte. Conducted with 11 mA g^−1^ (corresponds to a C‐rate of 0.1 C for [Na(2G)_*x*_]C_20_).

**Figure** **4** shows the ECD results over five cycles for a cell with EN as a co‐solvent. Compared to the results for pure 2G (see Figure 1), it can be clearly seen that the addition of EN effectively decreases the thickness change of the electrode. In the first cycle, the expansion decreases from 175% to 100%. In the second to fifth cycle, the electrode breathing is only 17–20% when EN is used compared to 46–49% with 2G as the only electrolyte solvent. This might be explained through changes in the overall cell reaction as well as the intercalation in the graphite structure of the smaller EN molecules compared to 2G molecules. Thus, the use of a suitable co‐solvent can be much more effective for the reduction of volume expansion than the variation of the type of glyme (compare to ref. [12]).

**Figure 5:**
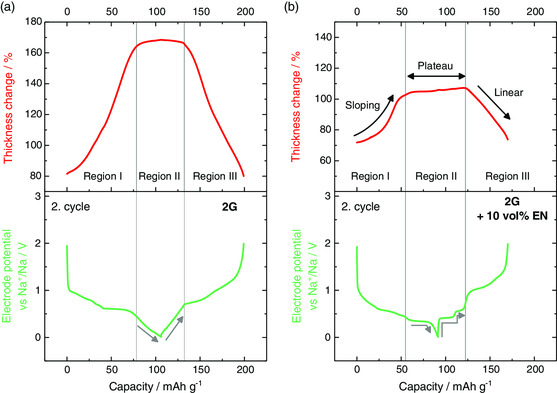
Comparison of the second cycle of the in situ ECD measurements for a) 2G and b) 2G + 10 v/v EN as electrolyte solvents. Three‐electrode cell set‐up with sodium as the counter and reference electrode and graphite as the working electrode. Conducted with 11 mA g^−1^ (corresponds to a C‐rate of 0.1 C for [Na(2G)_*x*_]C_20_). Regions I–III indicate segments of electrode expansion (I), constant electrode thickness (II) and electrode shrinkage (III). Gray arrows indicate the different voltage behavior in region II for both electrolyte solutions. Black arrows indicate the asymmetric ECD signal for the EN‐containing electrolyte.

**Figure** **5** shows the relative thickness change and the voltage profile for the second cycle of the cells made with pure 2G and the ones made with the addition of 10 v/v EN in more detail. The first cycle is shown in Figure S7, Supporting Information, but it was omitted due to the low ICE value. As mentioned earlier, the most notable impact of EN is the reduction in the electrode breathing and the higher complexity of the voltage profile. However, some additional similarities and differences can be seen. For more clarity, we discuss these by means of three regions (labeled I, II, and III); see Figure 5. First of all both systems show a sloping increase (region I) of the electrode thickness during sodiation followed by a plateau (region II). For 2G and 2G + 10 v/v EN, the storage capacities of the plateau regions are 28 and 36 mAh g^−1^, respectively, corresponding to fractions of 26% and 40% of the total discharge capacity. For cells with 2G as sole solvent, we recently assigned this plateau to a pseudocapacitive storage mechanism^[8a]^ because in this region, charge is being stored without any significant change in electrode volume and the voltage almost linearly changes during sodiation/desodiation (indicated by the gray arrows). In other words, in region II, the graphite structure seems to be temporarily pillared. The situation seems more complex in the case when EN is present. Then, region II during discharge correlates with a flat voltage plateau at ≈0.4 V, followed by a sharp voltage drop until the cut‐off voltage of 0.01 V (gray arrow). A flat voltage is typical for two‐phase reactions, i.e., it indicates a stage transformation. Still, the electrode expansion in this region is very small, which would support the suggestion by Zhang et al. that the graphite layers are pillared by the stronger interaction with the co‐intercalated solvent molecules.^[^ ^14^ ^]^ Second, the voltage profile and ECD signal are symmetric during sodiation/desodiation in case of 2G and they become asymmetric if EN is added. Although the electrode expansion in region I is sloping, the electrode shrinkage in region III is almost linear (indicated by the black arrows). Moreover, in region II, the single flat discharge voltage at ≈0.4 V splits into two plateaus during charging (see gray arrows). Such an asymmetric behavior for voltage and ECD signals is unexpected and indicates that the sodiation and desodiation are governed by different processes.

At the present, it is difficult to rationalize this asymmetric behavior and further studies are required to provide a convincing explanation. It is possible that the stronger interaction of EN within the t‐GIC compound influences the dynamic behavior of the electrode reaction. A hint toward the asymmetric behavior being influenced by kinetics can be seen from the voltage hysteresis plot shown in Figure S8, Supporting Information. Although the polarization is small in region II, the voltage profiles show that the polarization in case of EN increases with the degree of charging, which indicates that kinetic effects play a role. Nevertheless, the results clearly demonstrate that ECD is a very useful tool to study the electrode behavior during cycling through which important characteristic features can be identified.

Interestingly, the ECD results only partially agree with the ex situ XRD results from Zhang et al. Although Zhang et al. suggest that the use of EN as a co‐solvent along with 2G leads to a permanent pillaring (constant lattice expansion regardless of the state of change), our results indicate that pillaring occurs for 2G and 2G + EN and occurs only in a certain stoichiometry range (region II), i.e., the pillaring is only temporary, as breathing of the electrode during cycling is clearly detected. The temporary pillaring could also explain the findings by Zhang et al., who suggest that the degree of pillaring depends on the EN content. Nevertheless, the change in electrode breathing from 2G to 2G + EN is found to be similar, Zhang et al. see a reduction of 57% by ex situ XRD when EN is added, while a reduction of 61% can be found with in situ ECD. Considering our results and the results by Zhang et al., the behavior of t‐GIC in electrochemical cells with different solvent molecules is a largely unexplored research field that requires further systematic studies and the use of additional in situ methods.

## 3 Conclusion

ECD was used to study the influence of various parameters on the expansion/shrinkage (“breathing”) of graphite electrodes during intercalation of solvated sodium ions. Starting from the standard configuration, i.e., a graphite electrode containing PVDF as the binder, an electrolyte solution based on 2G, and sodium as the counter electrode, two strategies for reducing the electrode breathing were studied.

First, replacing the PVDF binder with CMC was only effective for significantly reducing the electrode expansion during the initial sodiation (175% vs 142%). No significant influence of the binder was found considering the breathing during subsequent cycles (46–49% for PVDF vs 45–55% for CMC). The beneficial properties of CMC were further confirmed for electrodes with different loadings (2.4–7.1 mg cm^−2^).

Second, the use of EN as a co‐solvent was evaluated. The addition of 10 v/v EN to 2G significantly reduces the initial electrode expansion by 43% (175% vs 100%). However, the use of EN also leads to more side reactions in the first cycle, resulting in an ICE value of only 38% compared to 74% without EN. On the other hand, the use of EN significantly reduces the degree or breathing over cycling. Although similar capacities are obtained for the EN and EN‐free cell, the breathing for the former is only 17–20% compared to 46–49% for the latter. Despite the fact that these are still large values, the results clearly show that adding co‐solvents can be an effective strategy to minimize electrode expansion/shrinkage during cycling. Moreover, the ECD results reveal clear differences in the storage mechanism between the 2G and 2G + EN electrolyte solution. The results also suggest a temporary pillaring of the graphite structure during which the electrode thickness remains largely constant (region II). Permanent pillaring does not seem to take place under the chosen experimental conditions.

## 4 Experimental Section

4.1

4.1.1

##### Electrode Preparation

The electrodes contained 90 w/w graphite powder (MTI Corp.) and 10 w/w binder material. NMP (Sigma‐Aldrich) was used as the solvent to cast electrodes when PVDF (PI‐KEM Ltd) was used as the binder. In the case of CMC (PI‐KEM Ltd), an aqueous solution was prepared. Electrodes were cast with different initial thicknesses to obtain different mass loadings and thicknesses and dried overnight in air. Finally, the electrodes were punched out and dried again under vacuum overnight at 110 °C.

##### Electrochemical Measurements

All cell assemblies took place in an argon‐filled glovebox from MBraun. Film thicknesses were measured inside the glovebox before cell assembly with a digital thickness dial gauge from Käfer Messuhrenfabrik GmbH. The sodium‐ion cells contained a graphite electrode with a diameter of 10 mm as the working electrode and pure sodium (BASF) as counter and reference electrode. 1 m NaPF_6_ (purity >99%, Sigma‐Aldrich) in 2G (Sigma‐Aldrich, pre‐dried using a 4 Å molecular sieve) was used as the electrolyte. The lithium‐ion cells contained the earlier‐mentioned graphite electrode as the working electrode and lithium (Rockwood Lithium) as counter and reference electrodes. 1 m LiOTf in 2G was used as the electrolyte. The in situ ECD experiments were conducted using an ECD‐nano cell device from EL‐CELL GmbH. The cell was designed as a three‐electrode set‐up. Working and counter electrode were separated by a fixed glass ceramic separator so that only the thickness change of the working electrode was measured. Around 250 μL electrolyte were used for one cell. The ECD experiments were conducted with a Biologic SP‐50 instrument at 25 °C. GCPL (galvanostatic charge and discharge with potential limitation) experiments were conducted between 0.01–2 V versus Na^+^/Na for the sodium‐ion cells and between 0.1–2 V versus Li^+^/Li for the lithium‐ion cells. The voltage window for the lithium‐ion cells was set to a higher lower cut‐off voltage, as a long voltage plateau occurred below 0.1 V, which might have been caused by side reactions.^[^ ^23^ ^]^ The thickness change in the electrodes was measured simultaneously with the GCPL experiments. For the ECD experiments with EN as a co‐solvent, the same electrodes as mentioned earlier for the sodium‐ion cell were used. The electrolyte consisted of 1 m NaOTf (purity >98.0%, Sigma‐Aldrich) in 2G + 10 v/v EN (Sigma‐Aldrich, pre‐dried using a 4 Å molecular sieve). NaOTf was purified before by dissolving in ethanol, reflux, hot filtration, and drying for 24 h at 120 °C under vacuum. Previous experiments showed that there is no notable impact on the electrode expansion/shrinkage between PF_6_^−^ and OTf^−^.^[^ ^15^ ^]^ The relative thickness changes (%) shown as red curves on Figure 1, 4, 5, S3, S4, S5, and S7, Supporting Information, were calculated by using

(2) $$h_{\text{relative}}/\%=\frac{h_{x}-h_{0}}{h_{0}}\times 100\%$$

where, *h*_*x*_ is the thickness of the electrode at each point and h_0_ is the initial thickness before the first discharge. The thickness changes in each half cycle, mentioned in the text and shown in boxes in the aforementioned graphs, were calculated using the following equations

(3) $$h_{\text{relative}}/\%=\frac{h_{\text{max}}-h_{n}}{h_{n}}\times 100\%$$

or

(4) $$h_{\text{relative}}/\%=\frac{h_{\text{max}}-h_{n+1}}{h_{n}}\times 100\%$$

where *h*_max_ is the maximum thickness of the electrode in one cycle, *h*_n_ is the thickness at the beginning of each cycle (local minimum), and h_*n*+1_ is the thickness at the beginning of the next cycle.

##### Physical and Chemical Characterization

The cross‐section images of the electrodes were taken with a SEM (PhenomProX, Phenom World) using an accelerating voltage of 10 kV (electrode with CMC) and 15 kV (electrode with PVDF). Cross‐section images were generated by cutting one electrode with scissors and using special sample holders. Energy‐dispersive X‐ray spectroscopy (EDX) images were taken with the same device using an accelerating voltage of 15 kV. XRD was performed using a D2 Phaser instrument from Bruker. The measurement was conducted with a Cu X‐ray tube (30 kV, 10 mA) between 5° and 40°, using a step width of 0.05°. Air‐tight sample holders with a copper plate were used for the measurement. Electrodes for XRD were retrieved from coin cells that were cycled for 3½ cycles at 0.1 C, i.e., the measurement was stopped at a fully discharged state. Disassembling of the cells and XRD sample preparation took place inside an argon‐filled glovebox.

## Conflict of Interest

The authors declare no conflict of interest.

## Supporting information

Supplementary Material
