# Supplementary material for: Strategies for Alleviating Electrode Expansion of Graphite Electrodes in Sodium‐Ion Batteries Followed by In Situ Electrochemical Dilatometry
Source: Energy Technol (Weinh). 2020 Nov 19;9(3):2000880. doi: 10.1002/ente.202000880 (PMC7988600; doi:10.1002/ente.202000880)
Supplement: Supplementary file 1 — Supplementary Material [file ENTE-9-2000880-s001.pdf]

Supporting Information

Strategies for Alleviating Electrode Expansion of Graphite Electrodes in Sodium-ion Batteries Followed by in situ Electrochemical Dilatometry.

Ines Escher[a], *Yuliia Kravets*[a], *Guillermo A. Ferrero*[a], *Mustafa Goktas*[a], *Philipp Adelhelm*[a,b,]*

[a] Humboldt Universität zu Berlin, Institut für Chemie, Brook-Taylor-Str. 2, 12489 Berlin

[b] Helmholtz-Zentrum Berlin, Joint research group Operando Battery Analysis, Hahn-Meitner-Platz 1, 14109 Berlin

*Contact: philipp.adelhelm@hu-berlin.de

Table S1. General advantages and disadvantages of PVDF and CMC.

<u>**PVDF**</u>		<u>**CMC**</u>	
**Advantages:**	[1]	**Advantages:**	
+ High chemical stability		+ Aqueous processing possible (non-critical, low cost, fast electrode drying)	[2]
+ High electrochemical stability		+ Low-cost	
+ Mechanically stable		+ Thermal stability	[3]
		+ Better adhesion between electrode film and current collector than if PVDF is used (strong hydrogen and chemical bonding)	[3] [1]
**Disadvantages:**		**Disadvantages:**	
- NMP for processing (hazardous, teratogenic, irritating, expensive)	[2a, 4]	- More hydrophilic (more moisture adsorption)	[4b]
- Expensive	[1, 3a, 5]	- Brittle	[8]
- Limited binding rate	[1, 3a, 5]		
- High thermal expansion rate	[1, 3a, 5]		
- Reaction with lithiated graphite and metallic lithium at elevated temperatures	[6]		
- Swells in organic solvent (e.g. ethylene carbonate, diethyl carbonate)	[7]		

a) PVDF is poly(vinylidene difluoride), CMC is sodium salt of carboxymethyl cellulose, NMP is N-methylpyrrolidone.

Table S2. Overview of different characteristics of graphite electrodes with PVDF and CMC as binders and different loadings of active material.

	PVDF 5.0 mg cm^{-2}	PVDF 6.5 mg cm^{-2}	CMC 2.4 mg cm^{-2}	CMC 7.0 mg cm^{-2}	CMC 7.1 mg cm^{-2}
Capacities 2.–5. cycle [mAh g^{-1}]	97–113	101–106	100–109	95–99	100–102
Capacities 2.–5. cycle [mAh cm^{-2}]	0.48–0.56	0.66–0.69	0.24–-0.26	0.66–0.70	0.72–0.73
Thickness change 1. cycle [%]	ca. 146	ca. 175	ca. 118	ca. 142	ca. 140
Thickness change 2.–5. cycle [%]	ca. 51–63	ca. 46–49	ca. 39–57	ca. 45–55	ca. 39–51
Initial thickness with current collector [µm]	70	85	49	97	109

a) Data obtained with a three-electrode cell set-up with sodium as counter and reference electrode.

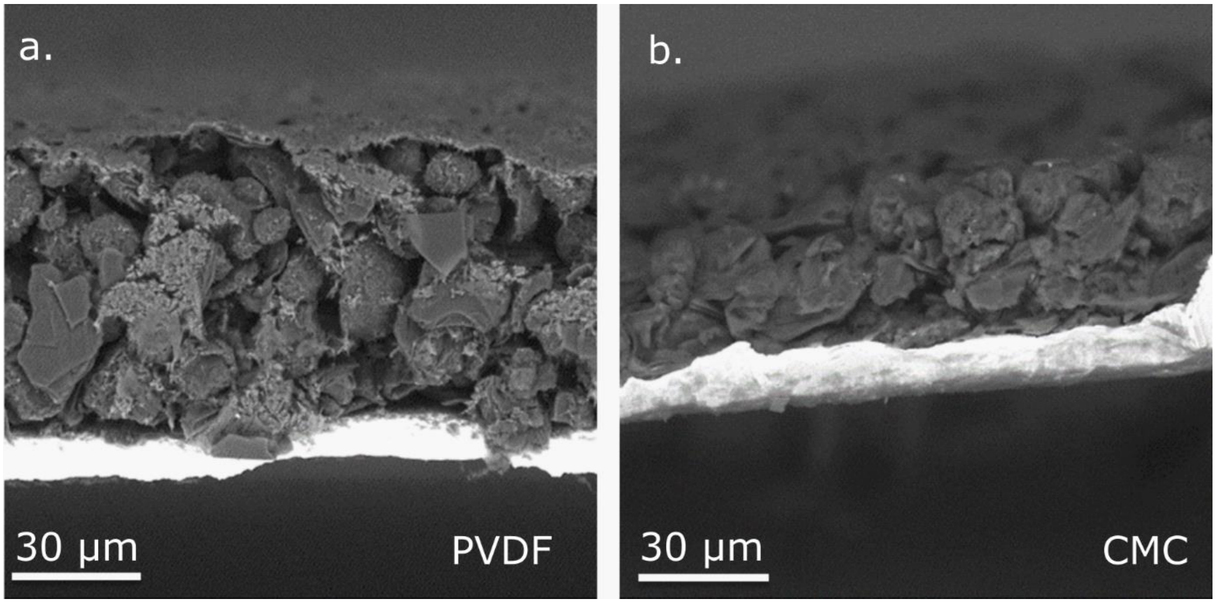

Figure S1. SEM cross section images of graphite electrodes made with 10 wt% PVDF (a.) and CMC (b.) binder. The shiny flat part on the bottom is the copper current collector.

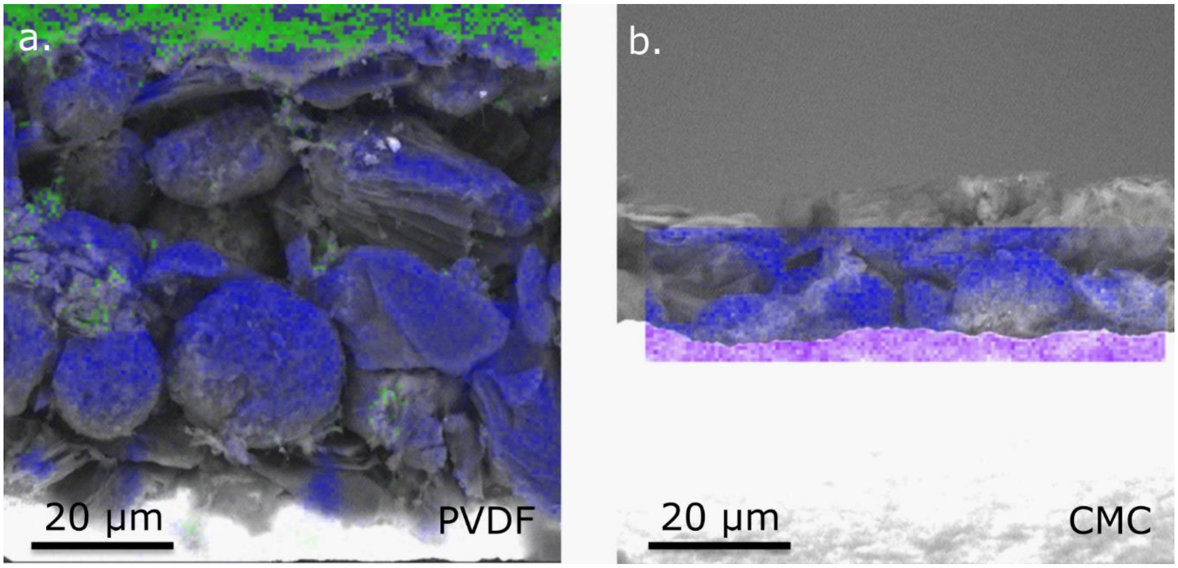

Figure S2. Elemental mapping of the cross section of graphite electrodes made with PVDF (a.) and CMC (b.). Blue = carbon, green = fluorine, purple = copper.

The graphite particles can be observed on the top, which are held together by the binder. In the case of PVDF, the binder can be clearly seen on the surface of the electrode (see Figure S1a). This is also confirmed by the elemental mapping displayed in Figure S2a and also shows that nearly no PVDF binder can be found in the bulk part of the electrode. In the case of CMC, the binder is much more homogeneously distributed within the electrode.

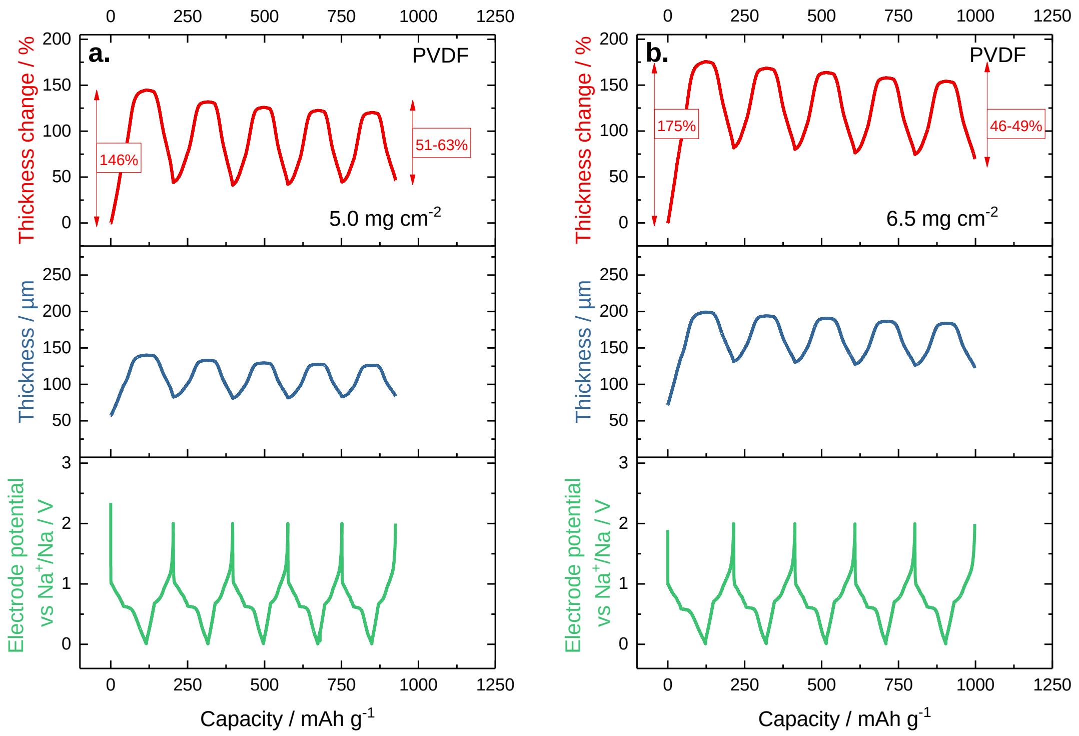

Figure S3. In situ electrochemical dilatometry experiments of graphite electrodes with PVDF as binder in in a three-electrode set-up with sodium as counter and reference electrode. Conducted with 11 mA g^{-1} (a.) and 10 mA g^{-1} (b.). Experiments shown with different electrode active mass loadings (a.: 6.5 mg cm^{-2}, b.: 5.0 mg cm^{-2}).

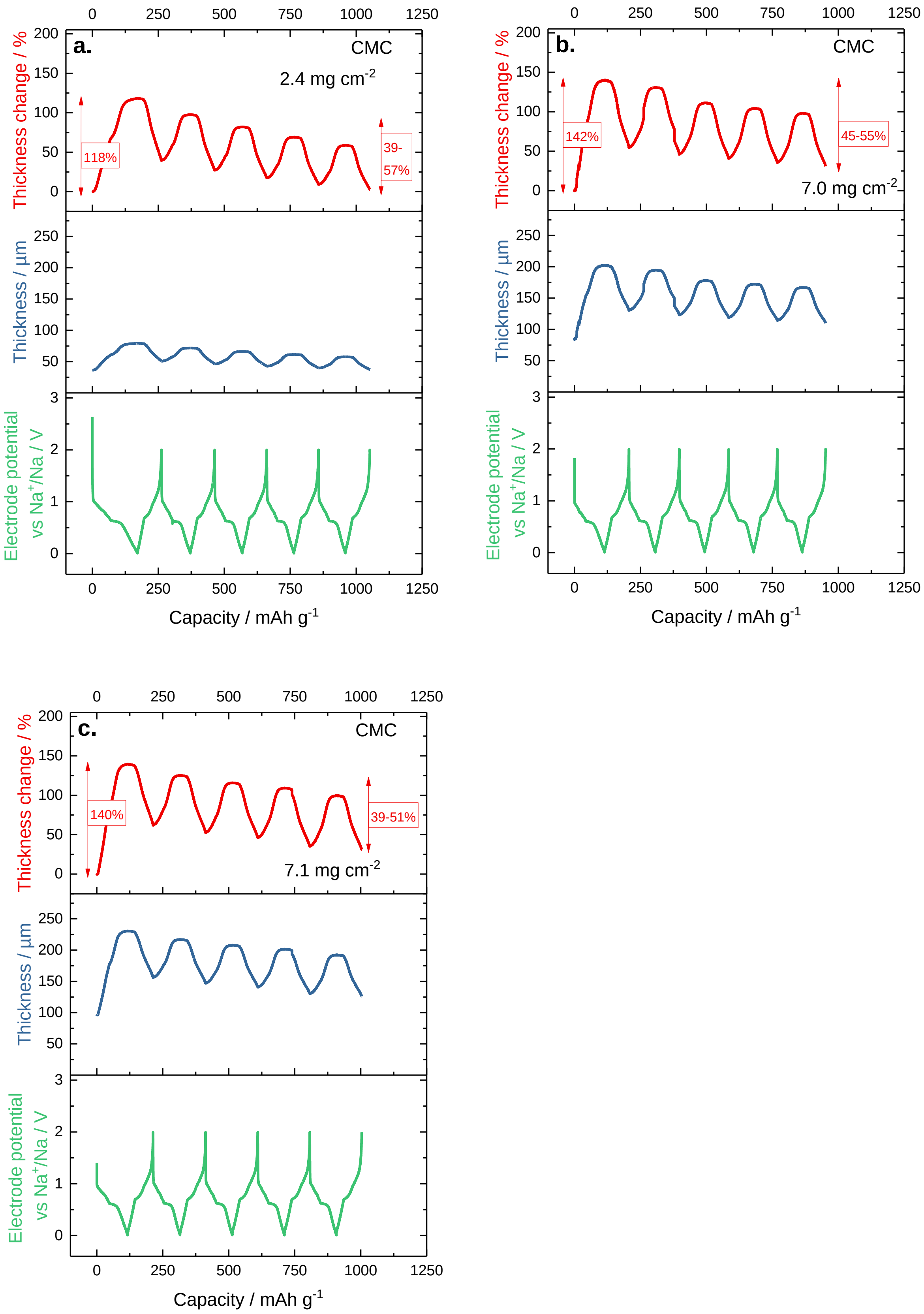

a.
CMC
2.4 mg cm-2
118%
39-57%
Thickness change / %
Thickness / µm
Electrode potential vs Na+/Na / V
Capacity / mAh g-1
b.
CMC
7.0 mg cm-2
142%
45-55%
Thickness change / %
Thickness / µm
Electrode potential vs Na+/Na / V
Capacity / mAh g-1
c.
CMC
7.1 mg cm-2
140%
39-51%
Thickness change / %
Thickness / µm
Electrode potential vs Na+/Na / V
Capacity / mAh g-1

Figure S4. In situ electrochemical dilatometry experiments of graphite electrodes with CMC as binder in a three-electrode set-up with sodium as counter and reference electrode conducted with a C-rate of 0.1 C (11 mA g^{-1}). Experiments shown with different electrode active mass loadings (a.: 2.4 mg cm^{-2}, b.: 7.0 mg cm^{-2}, c.: 7.1 mg cm^{-2}).

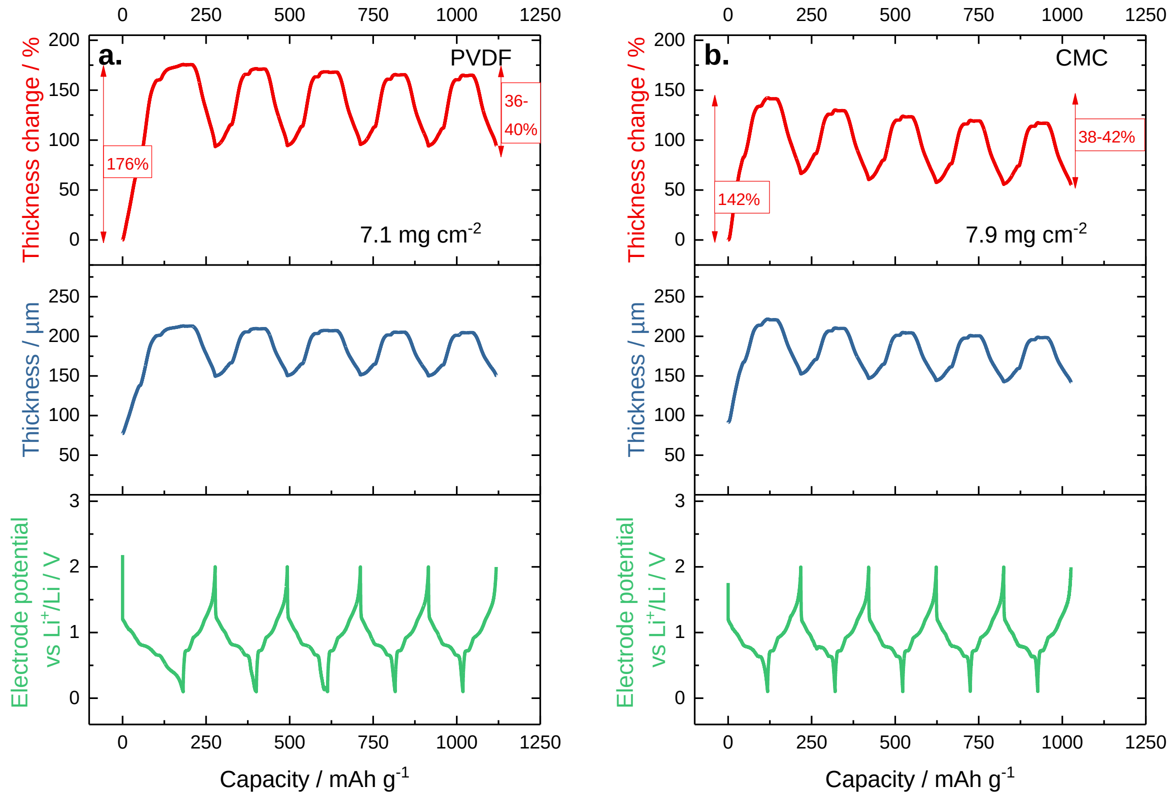

Figure S5. In situ electrochemical dilatometry experiments of graphite electrodes with two different binders (a. PVDF, b. CMC) in a three-electrode set-up with lithium as counter and reference electrode conducted with a C-rate of 0.1 C (11 mA g^{-1}).

The previous results are confirmed by performing similar experiments in lithium-ion half-cells. The results of these experiments are shown in the Figure S5. The voltage profiles is slightly more uneven compare to SIBs, a behavior known from the literature.[9] Regarding the binder influence, a similar trend was found as in the case of sodium-ion half-cells. Thus, the expansion in the first cycle is 19% smaller for the CMC electrodes compared to those made with PVDF (142% for CMC vs 176% for PVDF). In addition, the breathing of the electrodes in the following cycles is similar for CMC (38–42%) and PVDF (36–40%). It is worth to mention that for conventional LIBs, the total expansion of the battery should be limited to around 5%.[10] This, however, includes the whole cell system, i.e. expansion/shrinkage of both anode and cathode needs to be considered which can also compensate each other.

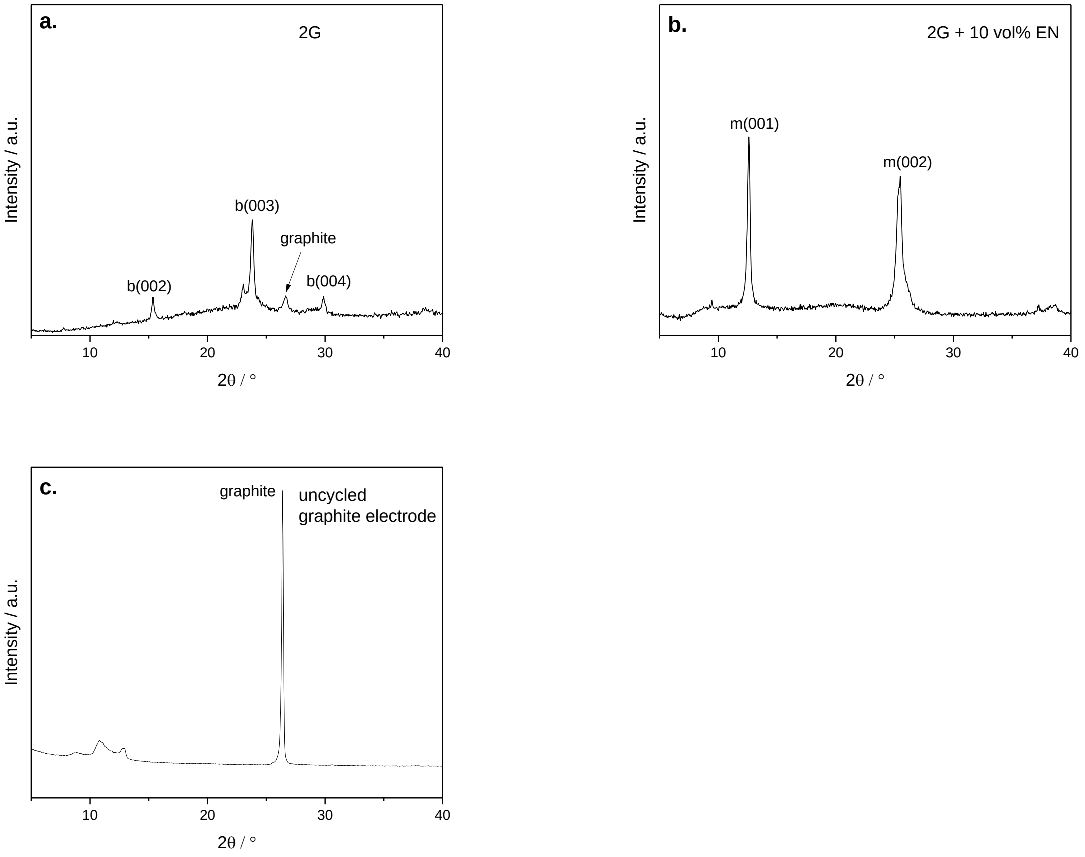

Figure S6. XRD pattern of a fully discharged graphite electrode with 2G (a.) and 2G + 10 v/v ethylenediamine (EN) (b.) as electrolyte solvent as well as an uncycled electrode (c.). Electrochemical experiments (a. and b.) conducted in a two-electrode set-up with sodium as counter electrode and a current of 11 mA g^{-1} (corresponds to a C-rate of 0.1 C for $[Na(2G)_x]C_{20}$). Labeling of the signals according to the results from Zhang et al.[11], where *m* indicates the monolayer t-GIC and *b* the bilayer t-GIC.

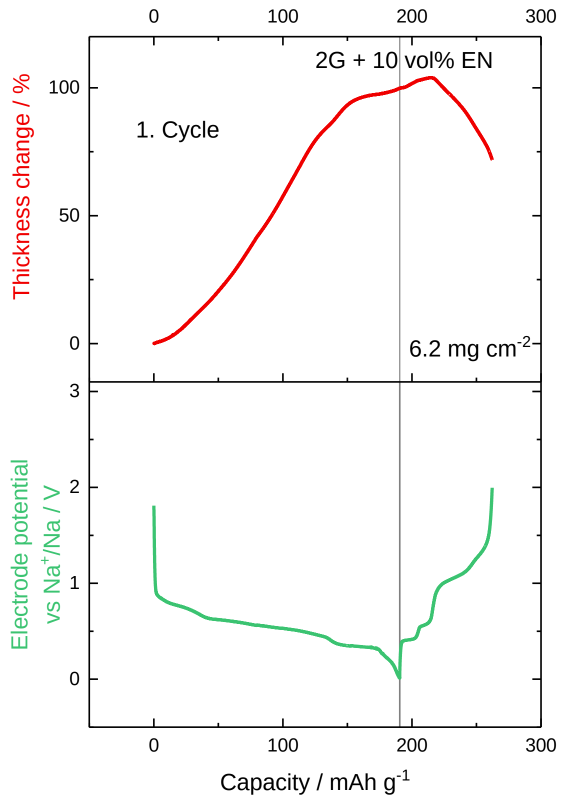

Figure S7. First cycle on an in situ electrochemical dilatometry experiments of a sodium-ion cell with graphite (PVDF as binder) as working electrode. 1 M NaOTf in 2G + 10 v/v EN is used as electrolyte. Three-electrode cell set-up with sodium as counter and reference electrode. Conducted with 11 mA g^{-1} (corresponds to a C-rate of 0.1 C for $[Na(2G)_x]C_{20}$).

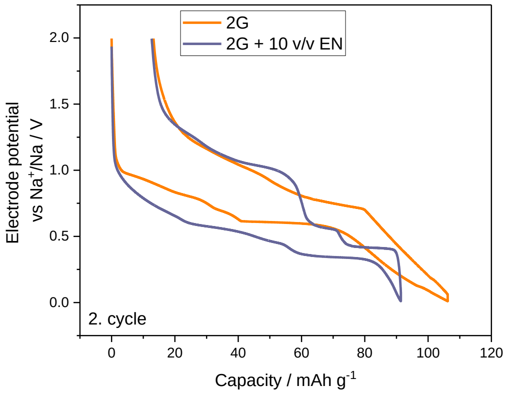

Figure S8. Voltage hysteresis plot (second cycle) of a sodium-ion cell with graphite (PVDF as binder) as working electrode in a three-electrode cell set-up with sodium as counter and reference electrode. Comparison of pure 2G as electrolyte solvent (orange) and the addition of 10 v/v EN (purple).

Comments on experimental challenges due to the use of ethylenediamine in electrochemical cells

Ethylenediamine (EN) is a highly reactive solvent and therefore electrochemical measurements in half cells were challenging. Pure EN cannot be used as solvent as it readily forms electrides and H_2 with sodium, which is used as counter and, or reference electrode. Due to that the surface of the sodium appears blue and H_2 gas evolves which could lead to a cell blow off.[11] The blue color can be seen in Figure S9.

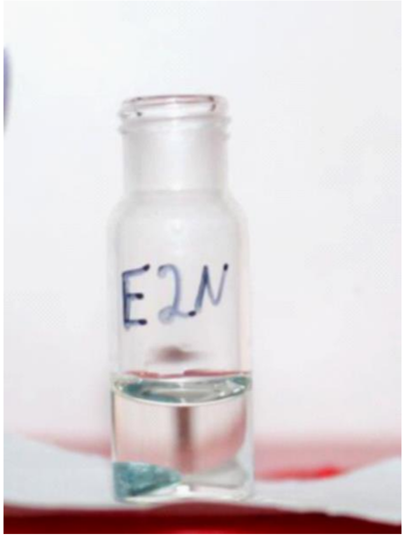

Figure S9. The picture shows a piece of sodium after 24 h of exposure to pure ethylenediamine.

References

[1] H. Chen, M. Ling, L. Hencz, H. Y. Ling, G. Li, Z. Lin, G. Liu, S. Zhang, *Chem. Rev.* **2018**, *118*, 8936.
[2] a) D. L. Wood III, J. Li, C. Daniel, *J. Power Sources* **2015**, *275*, 234; b) N. Susarla, S. Ahmed, D. W. Dees, *J. Power Sources* **2018**, *378*, 660.
[3] a) M. Valvo, A. Liivat, H. Eriksson, C.-W. Tai, K. Edström, *ChemSusChem* **2017**, *10*, 2431; b) M. Ling, Y. Xu, H. Zhao, X. Gu, J. Qiu, S. Li, M. Wu, X. Song, C. Yan, G. Liu, S. Zhang, *Nano Energy* **2015**, *12*, 178.
[4] a) F. M. Courtel, S. Niketic, D. Duguay, Y. Abu-Lebdeh, I. J. Davidson, *J. Power Sources* **2011**, *196*, 2128; b) D. L. Wood III, J. D. Quass, J. Li, S. Ahmed, D. Ventola, C. Daniel, *Drying Technol.* **2018**, *36*, 234.
[5] Z. Zhang, T. Zeng, Y. Lai, M. Jia, J. Li, *J. Power Sources* **2014**, *247*, 1.
[6] A. Du Pasquier, F. Disma, T. Bowmer, A. S. Gozdz, G. Amatucci, J.-M. Tarascon, *J. Electrochem. Soc.* **1998**, *145*, 472.
[7] Z. Chen, L. Christensen, J. R. Dahn, *J. Electrochem. Soc.* **2003**, *150*, A1073.
[8] J. Li, L. Christensen, M. N. Obrovac, K. C. Hewitt, J. R. Dahn, *J. Electrochem. Soc.* **2008**, *155*, A234.
[9] H. Kim, G. Yoon, K. Lim, K. Kang, *Chem. Commun.* **2016**, *52*, 12618.
[10] R. Dash, S. Pannala, *Mater. Today* **2016**, *19*, 483.
[11] H. Zhang, Z. Li, W. Xu, Y. Chen, X. Ji, M. M. Lerner, *Nanotechnology* **2018**, *29*, 325402.
